# Combining AI and new genomic techniques to ‘fine-tune’ plants: challenges in risk assessment

**DOI:** 10.3389/fpls.2025.1677066

**Published:** 2025-10-02

**Authors:** Matthias Juhas, Bernd Rodekohr, Andreas Bauer-Panskus, Christoph Then

**Affiliations:** ^1^ Testbiotech e.V., Munich, Germany; ^2^ Aurelia Foundation, Berlin, Germany

**Keywords:** new genomic techniques (NGT), genome editing, genetic engineering, genetically engineered plants, risk assessment, artificial intelligence, GMO regulation, cis-regulatory elements

## Abstract

Using new genomic techniques (NGTs) to ‘fine-tune’ plants typically involves changing just a small number of nucleotides. These small interventions can, nevertheless, lead to effects that go beyond the known plant characteristics, caused by genotypes previously unknown in the breeders’ gene pool. The EU is currently discussing a proposal for the future regulation of NGT plants. In essence, the European Commission is proposing that NGT plants with less than 20 deletions, insertions or substitutions should in future no longer undergo mandatory risk assessment. NGT plants up to this threshold would be classified as Category 1 NGT, and therefore treated as equivalent to conventionally-bred plants. Plants in this category would not be subject to mandatory environmental risk assessment. The question thus arises of whether any of these Category1 NGT plants considered, in fact, have novel environmentally hazardous characteristics. Based on our findings from horizon scanning and to exemplify regulatory challenges, we used publicly available generative AI with the aim to design ‘fine-tuned’ NGT plants that would very likely require environmental risk assessment, but would nevertheless meet the specific the criteria for Category 1 NGT plants. As a proof of principle, we designed a genetic blueprint for an insecticidal maize plant, which could subsequently be developed using NGTs. There are several reasons why these insecticidal NGT plants should be subject to environmental risk assessment prior to being approved for cultivation. For example, they could be toxic to non-target species, cause resistance in pest insects, or show unintended genetic and phenotypic changes. In summary, there is no scientifically justifiable threshold of a certain number of mutations up to which NGT effects could be assumed to be of the same category as conventionally bred plants. Therefore, it is essential that the future regulatory concept is not based on such thresholds. On the contrary, future regulation should be science based and include case-by-case and step-by-step risk assessment, traceability and monitoring requirements to secure the future of food production and to protect biodiversity.

## 1 Introduction

‘Fine-tuning’ plants with new genomic techniques (NGTs) primarily focuses on small but powerful regulatory genomic elements that are important for plant characteristics (Rodríguez-Leal et al., 2017). There is a growing trend to use artificial intelligence (AI) and large databases to enhance this strategy [see, for example (Li et al., 2024)]. Typically, the genetic alterations within this group involve changes to only a small number of nucleotides, but the effects may nevertheless exceed the range of known plant characteristics caused by genotypes previously unknown in the breeders’ gene pool [see, for example (Zhang et al., 2018)].

The EU is currently discussing a proposal for the future regulation of NGT plants. The European Commission has proposed the introduction of a threshold of not more than 20 genetic changes (of various types and size of mutations) in future regulation of plants obtained from new genomic techniques (European Commission, 2023). NGT plants with alterations up to this threshold would be categorised as NGT 1 plants. Any plants in this category [Annex 1 of (European Commission, 2023)] would be considered equivalent to conventionally-bred plants, and therefore not be required to undergo mandatory environmental risk assessment. If the criteria are met, equivalence would apply even if the properties of the NGT 1 plants were not known to exist in conventionally-bred varieties of the same species.

The question thus arises of whether ‘fine-tuned’ Category 1 NGT plants, which the EU Commission would see as equivalent to conventionally-bred plants, could have novel environmentally hazardous characteristics. To answer this question, we used horizon scanning (National Academies of Sciences, Engineering, and Medicine, 2020) to review the range of current applications applying NGTs to ‘fine-tune’ plants. This resulted in the identification of two particular types of applications, which subsequently became the focus of our research going forward. In this context, we also examined whether (and if so in what way) AI was mentioned in the design of the NGT systems.

Based on findings from the horizon scanning and in order to exemplify regulatory challenges, we used publicly available regenerative AI to design NGT plants that were likely to be insecticidal, but which would nevertheless meet the specific criteria for Category 1 NGT plants proposed by the EU Commission.

## 2 Horizon scanning: ‘fine-tuning’ NGT plants

The horizon scanning identified a larger group of NGT applications in plants that could technically be described as ‘fine-tuning’. The general concept involves choosing regulatory elements that can be targeted in genomic interventions to obtain effects which can be quantified and scaled (see, for example (Hou et al., 2022; Luo and Palmgren, 2023; Yadav et al., 2023). It is assumed that targeted genetic interventions which allow the intended effects to be scaled, may become more predictable and more balanced in regard to potential trade-offs (Rodríguez-Leal et al., 2017; Hou et al., 2022).

In many cases, even small genetic changes are sufficient to result in significant effects which may exceed the spectrum of known plant characteristics, both in quality and quantity. Examples include an altered sugar content (Xing et al., 2020), plant architecture (Tian et al., 2024), date of flowering (Zhou et al., 2024), resistance to stressors (Son and Park, 2023) and impact on yield (Song et al., 2022).

These applications share some common features:

- Key targets include regulatory genomic units that impact gene expression, e.g. promoters (Hou et al., 2022), upstream open reading frames (Luo and Palmgren, 2023) or miRNA (Yadav et al., 2023).
- Nucleases and transformation processes that are specifically tailored to achieve defined deletions (Zhou et al., 2023), or inversions (Schmidt et al., 2019), or to introduce newly designed short sequences (López-Dolz et al., 2022; Gupta et al., 2023).
- Large databases that allow the use of specific AI tools to identify the target regions and suggest the most effective genetic changes (Li et al., 2024).

Interventions in cis-regulatory elements (CREs), such as promoters or upstream open reading frames (uORFs), were found to be frequent targets for scaling various traits in NGT plants as described below. We selected the above two types of applications to exemplify the processes and outcomes of ‘fine-tuning’ in NGT plants.

Typically, the genetic alterations within this group involve changing only a small number of nucleotides. Therefore, we assume that the future regulation proposed by the EU commission would not require mandatory risk assessment for the majority of these NGT plants, even though, in most cases, the genetic changes appear to be unknown in the existing gene pool. It also appears to be the case that some of the technically-induced genetic changes would be very unlikely to emerge from non-targeted mutations, e.g. specific combinations of genetic changes within very small regions of the genome (Zhou et al., 2023, 2024). Therefore, many plants within this group are likely to substantially deviate from known conventionally-bred plants. Our findings are summarised below.

## 3 CRE as targets for NGT applications

**Figure 1:**
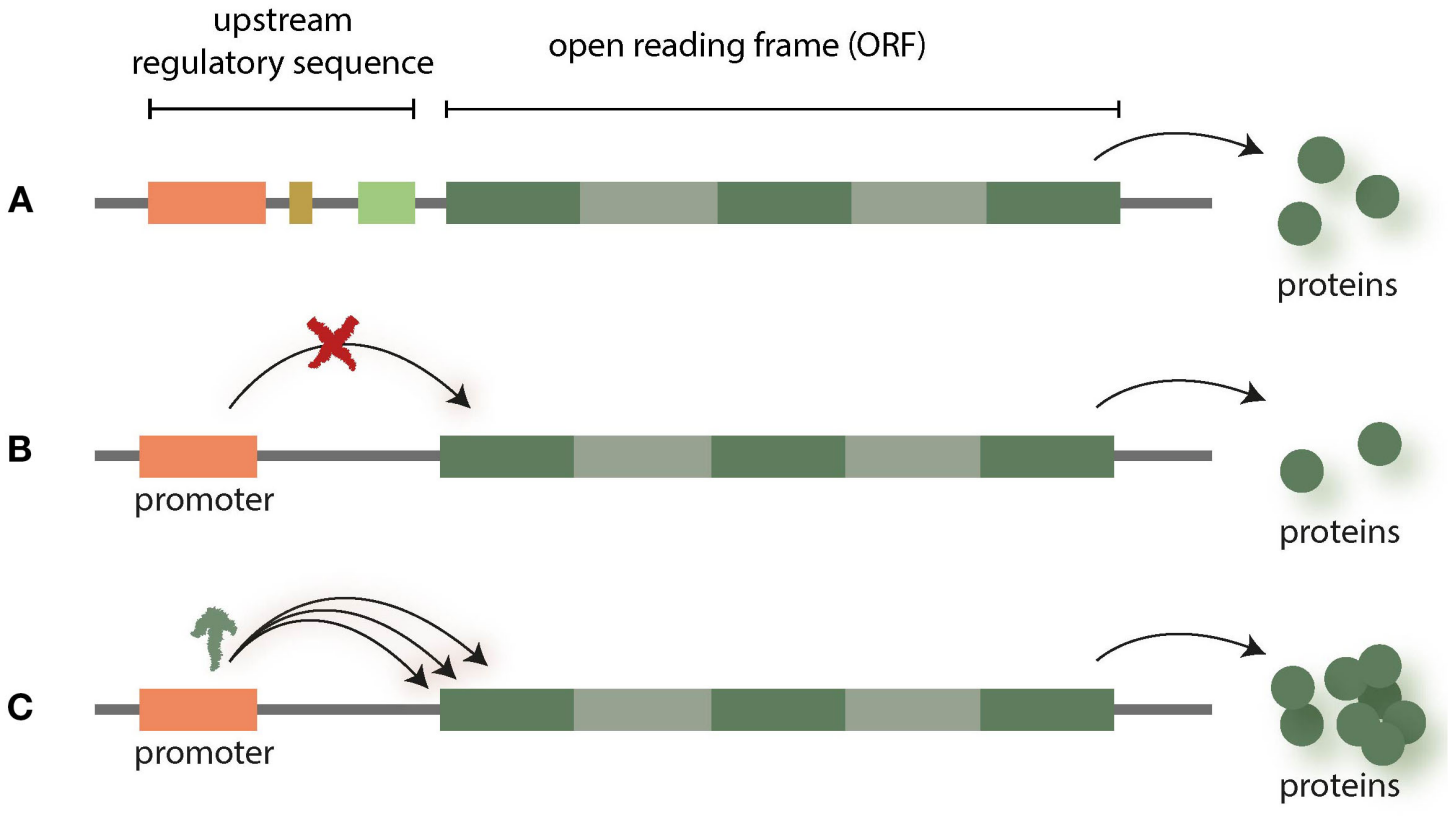
Promoters regulate the transcription of neighbouring genes **(A)**. The expression of respective genes can be altered (up- or downregulated) by interfering with promotors **(B, C)** [for overview see (Villao-Uzho et al., 2023)].

Cis-regulatory elements (CREs) are regions of non-coding DNA (enhancers, insulators, silencers) which regulate the transcription of neighbouring genes. In the context of NGT applications, promoters are one of the most interesting targets, as many of them are well characterized (Villao-Uzho et al., 2023). NGT research is now attempting to introduce changes within the promoters to achieve scalable effects in gene expression by reducing or enhancing the transcription of gene products (see **Figure 1**).

In regard to specific technical progress in this field, we found the work carried out by Zhou et al. to be especially significant (Zhou et al., 2023): previous research showed that, in many cases, arbitrary changes in nucleotides within the respective DNA sequences, e.g. those resulting from the use of CRISPR/Cas9, were not efficient enough to achieve the desired effects (Tang and Zhang, 2023). Therefore, for example, Zhou et al. applied a more recent type of nuclease known as CRISPR/Cas12a (Zhou et al., 2023). These nucleases enable the targeted deletion of nucleotides e.g. within the DNA of promoters, whereby this can be used to combine several targets in order to achieve the desired scalable effects in gene expression. By interfering with the gene expression of plant growth hormones, Zhou et al. were able to produce variations in rice plants, e.g. with reduced height (dwarf or semidwarf phenotypes), that showed no reduction in yield (Zhou et al., 2023). In addition to this example, there are several other NGT projects targeting plant promoters (Rodríguez-Leal et al., 2017; Hou et al., 2022; Tang and Zhang, 2023; Wu et al., 2024; Zhou et al., 2024; Wan et al., 2025). These employ several AI tools to focus on the identification and alteration of regulatory (promoter) elements in the plant genome (Levy et al., 2022; Deng et al., 2023; Li et al., 2023; Yasmeen et al., 2023).

## 4 uORFs as targets for NGT applications

**Figure 2:**
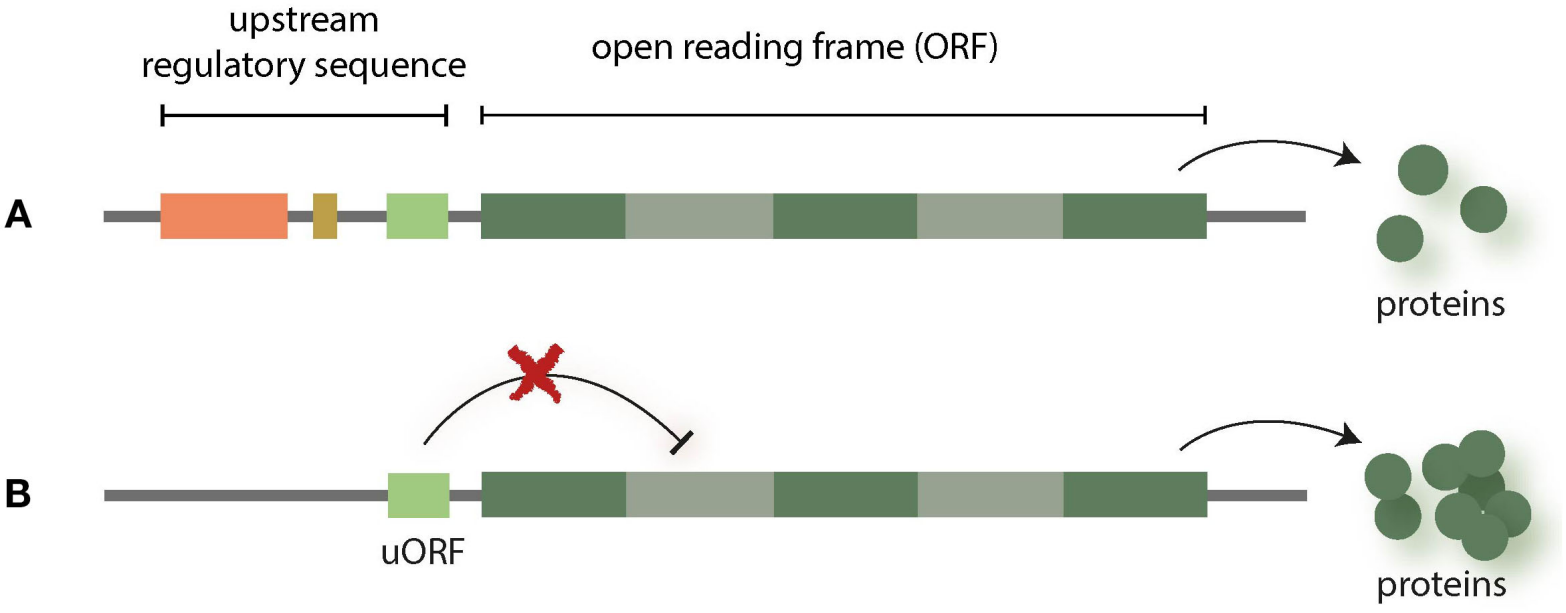
uORFs are small open reading frames (ORFs) that are upstream from the actual ORFs which are transcribed into messenger RNA (mRNA). uORFs can interfere with translation and thereby, e.g. reduce the production of related proteins [see (Tran et al., 2008)] **(A)**. Therefore, knocking out uORFs can be used to enhance the expression of specific genes **(B)**.

Upstream open reading frames (uORFs) are another emerging field of interest for NGT research. These regulatory elements are small open reading frames (ORF) located in the 5′ UTR and preceding the main ORF (mORF), which is transcribed into messenger RNA (mRNA). uORFs are based on DNA sequences that often are highly conserved and which interfere with or suppress the process of translation, thereby reducing the production of related plant proteins (Tran et al., 2008; Wang et al., 2024). Therefore, uORFs knockout with NGTs can be used to enhance the expression of specific gene functions (see **Figure 2**).

One of the first studies on targeting uORFs with NGTs was aimed at interfering with enzyme regulation. The outcome was an unprecedented 150 percent increase in ascorbate content in lettuce (Zhang et al., 2018). An increase in tolerance to the herbicide paraquat was also observed. Several other research teams are also using this approach of interfering with uORFs (Si et al., 2020; Um et al., 2021; Luo and Palmgren, 2023; Xue et al., 2023; Li and Wei, 2024). Furthermore, research is also underway to integrate additional short DNA sequences into uORFs in order to enhance or suppress gene expression (Xue et al., 2023).

Interventions in uORFs and promoters can also be combined with interventions in other regulatory units. For example, Guo et al. tried to change the heading date of rice by multi-target editing of the promoter and distal regulatory regions of two target genes (Guo et al., 2025). The US company genXtraits developed specific AI to identify uORFs for which it filed an international patent application (WO2023230631). Furthermore, specific databases are available with known uORFs in plants (Chen et al., 2020).

## 5 Planned EU regulatory framework for NGT plants

In 2023, the European Commission proposed a new and unique regulatory framework for NGT plants (Proposal 2023/411), which is still currently being discussed (European Commission, 2023). In Annex 1, the proposal includes threshold criteria that could allow the NGT plants to be approved without any requirements for specific GMO risk assessment. The plants would be categorised as NGT 1 plants and therefore considered to be equivalent to conventionally-bred plants with no requirements for environmental risk assessment. Neither would there be any requirements for methods of tracking and tracing the plants. Hybrid offspring produced by breeders, or emerging from spontaneous gene flow, would not be subject to any further assessment or approval processes.

The EU Commission is proposing a ‘threshold’ of not more than 20 genetic changes (European Commission, 2023). Each mutation may encompass alterations in up to 20 contiguous nucleotides or deletions and inversions without any size limitation. In addition, cisgenic DNA of unlimited length may be inserted. The EU Council proposed raising this threshold considerably by multiplying the 20 genetic changes in plant species with larger genomes (more than two sets of chromosomes), e.g. in hexaploid wheat (European Council, 2025). Our findings from horizon scanning show that almost all of the ‘fine-tuned’ NGT plants would fall within this category.

The proposal of the EU Commission states:

“*A NGT plant is considered equivalent to conventional plants when it differs from the recipient/parental plant by no more than 20 genetic modifications of the types referred to in points 1 to 5, in any DNA sequence sharing sequence similarity with the targeted site that can be predicted by bioinformatic tools.*

1. substitution or insertion of no more than 20 nucleotides;
2. deletion of any number of nucleotides;
3. on the condition that the genetic modification does not interrupt an endogenous gene:
  - targeted insertion of a contiguous DNA sequence existing in the breeder’s gene pool;
  - targeted substitution of an endogenous DNA sequence with a contiguous DNA sequence existing in the breeder’s gene pool;
4. targeted inversion of a sequence of any number of nucleotides;
5. any other targeted modification of any size, on the condition that the resulting DNA sequences already occur (possibly with modifications as accepted under points (1) and/or (2)) in a species from the breeders’ gene pool”.

The above criteria will be of major relevance to all companies wanting to benefit from fast-tracked marketing approvals in the EU. If the criteria are met, equivalence applies even if the properties of the NGT 1 plants are novel and do not occur in conventionally-bred varieties of the same species. Therefore, these criteria could be used as a ‘design room’ to develop plants with new characteristics that would not be subject to mandatory risk assessment (Vogel, 2025). This may be advantageous for the applicants, but it may also allow hazardous NGT plants (including wild plants) to be released into the environment without sufficient control.

## 6 Identifying strategies using AI to develop NGT 1 plants

In order to explore possible regulatory challenges and to test the ‘design room’ identified by Vogel (Vogel, 2025), we used the publicly available AI ChatGPT 4.o (deep research agent) to design a ‘technically tuned’ NGT maize plant by interfering with regulatory elements (cis-regulatory elements (CREs) and upstream open reading frames (uORFs)), which would essentially need to be subject to environmental risk assessment, but which could, in fact, be categorised as NGT 1. We chose an insecticidal trait for this small-scale ‘proof of concept’ experiment.

One approach suggested by ChatGPT was to enhance the expression of the endogenous gene coding for maize serine protease inhibitor (SPI), thus inducing insecticidal properties in NGT maize. SPIs are proteins that occur naturally in plants where they have a crucial role in protecting them against insect and pathogen infestation. If maize plants are attacked by pest insects, their natural defence mechanisms may be increased, but only for a short period of time, i.e. transiently (Chen et al., 2024). Insects feeding on the plants may take up SPIs, thus inhibiting the activity of certain enzymes they need to digest the plant material. As a result, the insects are no longer able to digest the food properly, which delays their development and leads to increased mortality. Insects belonging to the order of *Lepidoptera* are particularly susceptible in this respect. Several species from this group, such as *Ostrinia* spp, are considered to be pest insects in maize (Chen et al., 2024).

Research into ways of enhancing the content of SPI in plants to protect them more efficiently from pest insects has been ongoing for several years. Plants with an elevated content of SPI have already been produced with the help of transgenes (Clemente et al., 2019). However, the aim in this case was to use a publicly available AI chatbot to design an insecticidal NGT maize with increased SPI content, without transferring any genes from other species.

In a first attempt, the AI chatbot suggested replacing the natural promoter of the SPI gene, which is only transiently active, by a promoter that would cause constitutive expression of the target gene and would be part of the maize genome. As the promoter belongs to the same species, the resulting plant may be considered cisgenic and not transgenic. This strategy is based on planned future EU regulation, as both the insertion of cisgenic elements and the deletions of the native plant genes may meet the criteria for so-called NGT 1 plants. However, doubts were raised within our research group about whether the combination of a promoter in isolation with another gene would be generally accepted as cisgenesis. We could not find a clear definition in the EU Commission proposal to find an answer to this question. Therefore, we believe it is unclear whether this strategy is acceptable for developing NGT 1 plants under the current version of the draft regulation.

In a further attempt, several small point mutations were introduced into the native promoter of the SPI gene via base editing. Several regulatory elements were identified within the native promoter as naturally inhibiting the expression of the SPI gene. The ‘knock-out’ of these inhibitory regulatory elements can be expected to enhance expression of the SPI genes. Such limited genetic changes to the genome via multiplexing would certainly meet the requirements of Category 1 NGT plants – they are also likely to cause similar effects as the replacement of the promoter.

In maize, there are several genes of this special SPI group (more precisely: subtilisin-chymotrypsin inhibitors) from which five can be considered as natural variants and one as a gene copy of the target SPI gene (see **Supplementary Material 1**). In this ‘proof of concept’ experiment, the gRNA design and editing strategy of ChatGPT was aimed only at the target SPI gene, which could, however, also lead to unintended feedback effects within the SPI gene family. Depending on the actually resulting plant characteristics, for example, up-regulation of the other SPI group members may also be a possible further scenario within the ‘design room’ of the Commission proposal.

## 7 An in-silico experiment to develop insecticidal Category 1 NGT plants with the help of AI

### 7.1 General strategy

The ChatGTP 4.o ‘deep research’ function was used for all prompts. The ChatGPT 4.o proposals for designing the NGT plants provided by the ‘deep research’ function were not always successful, but could be corrected and improved by manual input. All the suggested genomic sequences, binding sites, gRNAs, cleavage-sites etc. were manually checked for correctness/integrity using NCBI (https://www.ncbi.nlm.nih.gov/gene), Maize Genetic and Genomic Database (https://maizegdb.org/), BLAST (https://blast.ncbi.nlm.nih.gov/Blast.cgi) and Clustal Omega (https://www.ebi.ac.uk/jdispatcher/msa/clustalo?stype=dna) for sequence alignment.

#### 7.1.1 Prompt

Prompt:

*Search for strategies to develop an insecticidal maize plant according to the NGT1 criteria of the EU proposal 2023/411, with a focus on effective protection against Lepidoptera. Consider both classical and cis-regulatory genome editing approaches and check if suitable resistance factors are available within the breeding pool*.

ChatGPT generated a short background and an objective summary followed by a classification of the proposed project in regard to the Category 1 NGT criteria in the EU proposal 2023/411 (EU Commission 2023). It provided suggestions regarding endogenous defence mechanisms against insects in maize and several target genes to increase plant resistance to lepidoptera. This included suggesting implementation and feasibility strategies.

One of the proposed target genes was an insecticidal serine protease inhibitor (SPI), for which the chatbot came up with several strategies to increase SPI gene expression. Exchanging the native SPI promoter with stronger or constitutive endogenous promoters (‘Promoter-Swap’) or inactivating ‘repressory’ regulatory elements of the SPI gene were amongst these strategies. Accordingly, these two strategies, i.e. i) ‘Promoter-Swap’ and ii) inactivation of ‘repressory’ regulatory elements were pursued further.

### 7.2 ‘Promoter-Swap’

#### 7.2.1 Prompt

*Create a gRNA design and editing strategy for: Production of an insecticidal NGT 1 maize plant (toxic to Lepidoptera) in which the SPI gene shows a permanently high expression pattern in aboveground plant parts by replacing the native SPI promoter with the endogenous 1.) strong promoter I and 2.) constitutive promoter II. The NGT 1 maize plant must meet the requirements of the EU Commission proposal 2023/411 Annex 1*.

**Figure 3:**
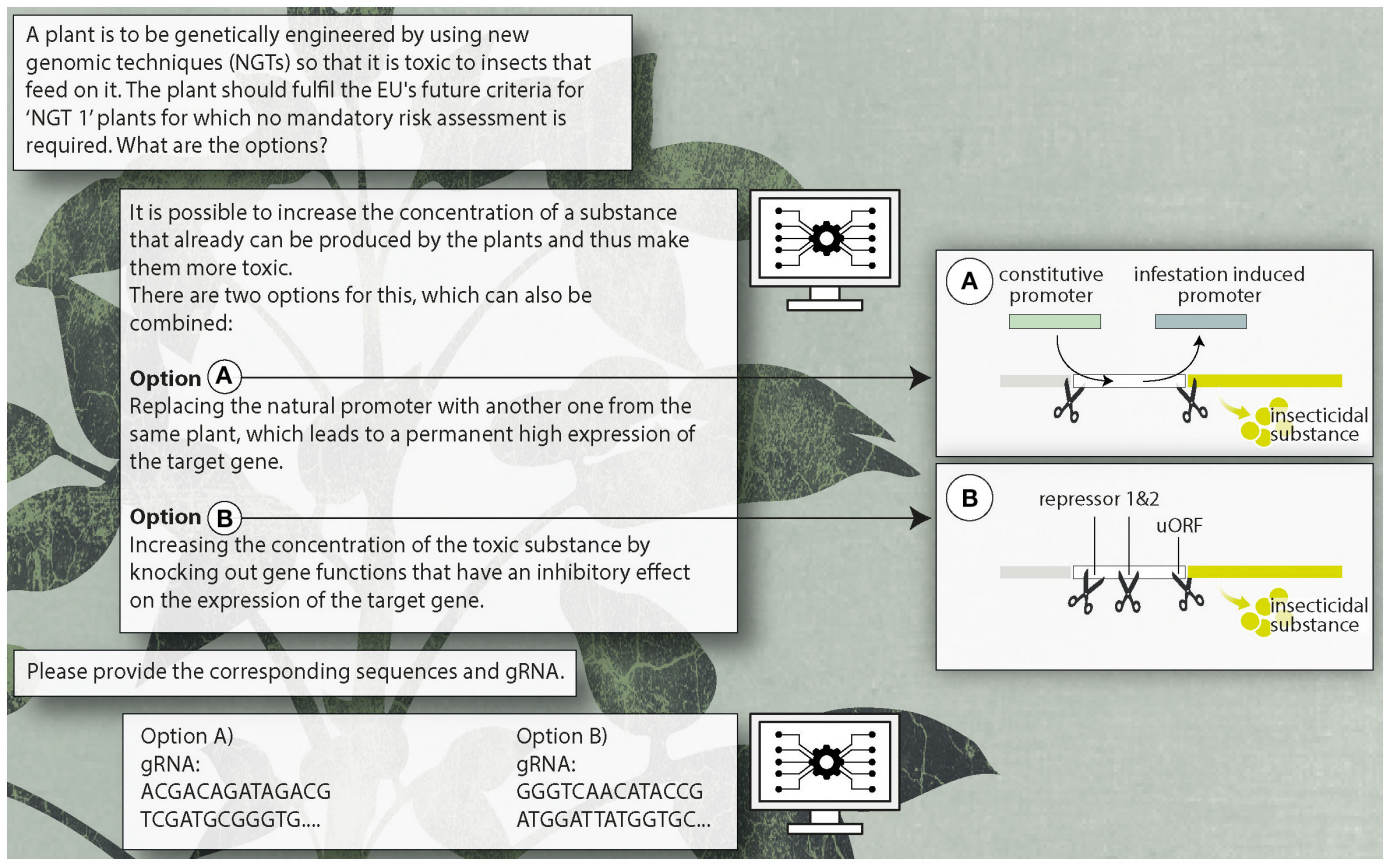
Overview of technical steps for the design of insecticidal NGT plants (DNA sequences in the diagram represent arbitrary combinations without function).

This provided a ‘promoter swap’ gene editing strategy for a constitutive and stronger promoter, using CRISPR/Cas9, a donor repair template (‘Promoter-Swap’ Cassette) for homology directed repair (HDR), suitable ‘guide’ RNAs and corresponding Cas9 cleavage sites (**Figure 3** Option A).

### 7.3 Inactivation of inhibitory regulatory elements

#### 7.3.1 Prompt

*Identify known cis-regulatory elements of the SPI gene in maize and propose point mutations using CRISPR-Cas9 to knock out repressive regulatory elements, resulting in significantly higher SPI expression.*

Several ‘repressory’ regulatory elements of the maize SPI gene were identified for subsequent inactivation, from which binding-sites for two different repressive transcription factors and one start-codon of an upstream open reading frame (uORF) were chosen as target sites. For inactivation of these three regulatory elements, point mutations via base editing (CRISPR/Cas coupled with a deaminase) was suggested. Again, this provided a strategy which included the identification of the individual nucleotides of the corresponding CREs, appropriate base editors and suitable gRNAs (**Figure 3** Option B).

It seems the genotypes and phenotypes of the in-silico plants were previously unknown and, at least in combination, unlikely to be obtained from conventional breeding, including random mutagenesis. Although there is no experimental data to compare the level of the SPI gene expression of the in-silico NGT 1 maize to its natural variations, it is plausible that this strategy could result in a constitutively higher gene expression and increased concentration of SPI in the NGT 1 maize plants.

For further details see **Supplementary Material 2** and **Figure 3**.

## 8 Need for risk assessment

Earlier attempts to enhance the gene expression of SPI via transgenic elements had some success (Clemente et al., 2019). However, mandatory risk assessment and labelling would be required for these transgenic plants before they could be released or marketed. This would not be the case with the above-described NGT plants, even if they contain a similar or higher concentration of the SPI protein compared to the transgenic plants. Therefore, some scientists have suggested using NGTs instead of transgenic techniques to produce plants with higher content of SPI, as these would escape mandatory risk assessment and make it easier to bring them to the market (Clemente et al., 2019).

There are however several reasons why these insecticidal NGT plants should undergo environmental risk assessment prior to being approved for cultivation. Fundamentally, the targeted regulatory elements are part of a fine-tuned network of interdependency within the genome and the cells, allowing the plants to defend themselves against pest insects. These networks have evolved through natural processes and are also involved in the response of plants to other environmental factors, e.g. pathogens and climate change, or interactions with other species, such as pollinators and soil organisms (Chen et al., 2024). These interdependencies have arisen through evolutionary processes and may be severely disturbed or disrupted by certain technical interventions. ‘Out-of-tune plants’ could seriously damage interconnected ecosystem functions, food webs and biodiversity. These risks also are relevant to plants with increased levels of SPI (Mangena, 2022).

Furthermore, plant health and food security may be seriously impaired if the plants can no longer respond to, or interact with, the environment as they have evolved to do. For example, if exposed to other pathogens and/or more extreme climate conditions, the ‘out-of-tune’ plants may drastically outperform others, as has already been seen with transgenic plants (Zeller et al., 2010).

Therefore, the risks AI-designed plants pose to the environment need to be examined on several levels:

### 8.1 Toxicity

There are several insect larvae, such as those belonging to *Ostrinia* spp, that are considered to be pest insects. However, more research is needed to assess the impact of plants with permanently high levels of SPI proteins on non-target insects (including those feeding on the pollen), the food webs as well as interactions with soil organisms and food production (Mangena, 2022).

### 8.2 Unintended effects

NGTs are also known to potentially cause unintended effects in the genome and the phenotype (Koller and Cieslak, 2023). For example, plant metabolism may be altered and cause changes in plant composition, seed viability and plant fitness. This can have negative consequences for food safety and the environment (ANSES, 2024).

### 8.3 Resistance in pest insects

A permanent certain concentration of SPI in NGT plants may allow some pest insects to adapt rapidly, and thereby weaken the natural defence mechanisms of conventionally-bred plants. Similar effects have been observed in the cultivation of transgenic insecticidal plants (Tabashnik et al., 2023).

### 8.4 Risks of gene flow

The appearance of teosinte in Spain and France (Trtikova et al., 2017) shows that there is some risk of gene flow from genetically engineered maize resulting in viable hybrid offspring, which could have characteristics absent in the parental plants (Arias-Martín et al., 2024) (EFSA, 2024).

### 8.5 Non-specified risks in regard to ecosystem functions and food production

The effects of plants on the food web, or their interactions with microbes and insects, are based on co-evolution that allows the ecosystem to maintain and further develop its function. Similarly to transgenic plants, NGT 1 plants can add characteristics to plant populations that go beyond the typical characteristics of these species (Kawall, 2021; Koller et al., 2024).

### 8.6 Cumulative risks

Depending on the speed of development, the depth of intervention and the scale of NGT organisms being introduced into the environments, tipping points may be reached that disturb or disrupt the stability of the ecosystem functions (Koller et al., 2023).

## 9 Discussion

As our example shows, generative AI can be used to design Category 1 NGT plants that meet the proposed criteria to avoid risk assessment, but they may still pose serious risks to health and the environment. However, there is much more to come: Generative AI tools able to identify numerous target regulatory units in the genome or generate options for genetic alterations and new gene combinations could considerably speed up the development of other NGT 1 plants with similar or even greater risks (Li et al., 2018, 2024; Zhao et al., 2021; Levy et al., 2022; López-Dolz et al., 2022; Deng et al., 2023; Kuang et al., 2023; Yasmeen et al., 2023; Daniel Thomas et al., 2024; Vogel, 2025).

Several companies are already using AI to develop NGT plants. It has to be assumed that their algorithms are much more effective than the publicly available version of ChatGPT. While a lot of discussion is centred around AI potentially being abused for evil purposes causing, e.g. major biosecurity risks, this example demonstrates that there can also be risks to biosafety. It shows that as far as NGT 1 plants are concerned, more attention must be paid to the protection of biodiversity and food production.

It should also be noted that other ‘fine-tuned’ NGT plants listed in the examples above would require risk assessment, as at least some of the scalable effects are likely to impact plant composition, fitness and interactions in the environment (Kawall, 2021). Furthermore, ‘fine-tuned’ NGT plants are not the only NGT 1 plants to be associated with environmental risk. NGT 1 plants with changes in plant composition (Kawall, 2021; Koller et al., 2024), earlier first date of flowering (Ortega et al., 2023), increased fitness (Koller et al., 2024) or changes in the interaction between plants and plant microbes (Yan et al., 2022), are further examples that need case-by-case in-depth risk assessment. Finally, unintended genetic changes also need to be considered in this context (Koller and Cieslak, 2023) as well as unexpected effects in the phenotype caused by e. g. feedback loops, epistasis or pleiotropy that only may be discovered upon closer examination of the NGT 1 plants.

As a result, the proposal made by the European Commission is not adequate to guarantee safe handling of NGT plants if they are released into the environment or brought to market. The potential to damage human health and the environment may increase over time, as an ever increasing number of Category 1 NGT plants would be approved for cultivation and/or import into the EU, without these having undergone risk assessment or their potential interactions. There would also be no possibility of checking the genetic stability of the plants’ (hybrid) offspring, e.g. in changing environmental conditions.

Our findings are supported by a recent study carried out by experts at the German Federal Institute for Nature Protection (BfN). This study concludes that artificial intelligence (AI) can be used to generate very complex biological effects, even if the number of changes remains below the proposed threshold. Biological changes could include the production of new proteins, or potentially insecticidal molecules or changes in gene regulation (Mundorf et al., 2025). It further concludes that the EU Commission proposal is neither scientifically justifiable nor suitable to adequately address the potential risks associated with NGT plants.

## 10 Recommendations

In summary, a threshold of a certain number of mutations to assume risk-free NGT effects is pointless. Therefore, a regulatory concept without any such thresholds is needed. Future regulation should be science based and include case-by-case risk assessment, traceability and monitoring to secure the future of food production and to protect biodiversity.

In this regard, ANSES could become a starting point to develop adequate regulatory concepts for the future regulation of NGT plants (ANSES, 2024). ANSES proposes a decision tree that works within current EU GMO regulation, but which can be used to adapt current risk assessment to the evolving field of NGT plants. It requires step-by-step and case-by-case risk assessment that can actually be fast-tracked if there are no indications in the first stages of reasons for concern. The applicant still has to provide some data, but the overall amount of data requested could be significantly reduced. This approach also allows to take into account unintended genetic changes and unexpected effects on the level of the phenotype.

Therefore, the concept as proposed by ANSES corresponds to the intentions of the EU Commission in its attempts to simplify the approval process for lower risk NGT plants (ANSES, 2024). At the same time, the regulation would still allow the political decision-maker to require data for risk assessment as well as to control and monitor the environmental releases of all NGT plants.
