# Supplementary material for: Combining AI and new genomic techniques to ‘fine-tune’ plants: challenges in risk assessment
Source: Front Plant Sci. 2025 Oct 2;16:1677066. doi: 10.3389/fpls.2025.1677066 (PMC12528024; doi:10.3389/fpls.2025.1677066)
Supplement: Supplementary file 1 [file DataSheet1.pdf]

<u>Supplementary material 1</u>

Annotated subtilisin-chymotrypsin inhibitors in *Zea mays* at NCBI (https://www.ncbi.nlm.nih.gov/gene). The target SPI gene of the small-scale proof of concept experiment is LOC100282452 (**bold**) and the possible gene copy is LOC100280933.

Gene ID	Description	Location	Aliases
LOC100278126	uncharacterized LOC100278126	Chromosome 8, NC_050103.1 (137953415..137953953)	-
LOC100280776	subtilisin-chymotrypsin inhibitor CI-1C	Chromosome 8, NC_050103.1 (95679956..95680387, complement)	ZEAMMB73_Zm00001d010025, GRMZM2G058358
LOC100280933	subtilisin-chymotrypsin inhibitor CI-1B	Chromosome 6, NC_050101.1 (47348992..47349691)	ZEAMMB73_Zm00001d035684, GRMZM2G000326
**LOC100282452**	**subtilisin-chymotrypsin inhibitor CI-1B**	**Chromosome 6, NC_050101.1 (47319217..47319828)**	**ZEAMMB73_Zm00001d035683, GRMZM2G096680**
LOC100286271	uncharacterized LOC100286271	Chromosome 6, NC_050101.1 (47122295..47122964)	ZEAMMB73_Zm00001d035680, GRMZM2G012928, si605061e03b
LOC100286333	subtilisin-chymotrypsin inhibitor CI-1B	Chromosome 6, NC_050101.1 (47126504..47127154)	ZEAMMB73_Zm00001d035681, GRMZM2G012806
LOC542408	subtilisin-chymotrypsin inhibitor homolog 1	Chromosome 8, NC_050103.1 (138036912..138037684)	ZEAMMB73_Zm00001d011080, GRMZM2G028393, MPI, sci1
