# Supplementary material for: Combining AI and new genomic techniques to ‘fine-tune’ plants: challenges in risk assessment
Source: Front Plant Sci. 2025 Oct 2;16:1677066. doi: 10.3389/fpls.2025.1677066 (PMC12528024; doi:10.3389/fpls.2025.1677066)
Supplement: Supplementary file 2 [file DataSheet2.pdf]

Supplementary material 2

Since the dialogue with ChatGPT took place in German, the conversation was translated for the manuscript supplement with DeepL (https://www.deepl.com/translator).

1) Identification of possible strategies for the design of insecticidal NGT-1 plants

Prompt:

I want to develop an insecticidal NGT plant according to the NGT1 criteria in EU Proposal 2023/411. The specifications are set out in Annex 1: https://food.ec.europa.eu/system/files/2023-09/gmo_biotech_ngt_proposal_2023-411_annex_en.pdf).

The NGT1 criteria are:

ANNEX I
Criteria of equivalence of NGT plants to conventional plants
A NGT plant is considered equivalent to conventional plants when it differs from the recipient/parental plant by no more than 20 genetic modifications of the types referred to in points 1 to 5, in any DNA sequence sharing sequence similarity with the targeted site that can be predicted by bioinformatic tools.

(1) substitution or insertion of no more than 20 nucleotides;
(2) deletion of any number of nucleotides;
(3) on the condition that the genetic modification does not interrupt an endogenous gene:
  (a) targeted insertion of a contiguous DNA sequence existing in the breeder's gene pool;
  (b) targeted substitution of an endogenous DNA sequence with a contiguous DNA sequence existing in the breeder's gene pool;
(4) targeted inversion of a sequence of any number of nucleotides;
(5) any other targeted modification of any size, on the condition that the resulting DNA sequences already occur (possibly with modifications as accepted under points (1) and/or (2)) in a species from the breeders' gene pool.

The strategy may be aimed at modifying cis-regulatory elements, but if there are more effective strategies, I am of course also interested in these. The insecticidal NGT1 plant should at best be a crop plant, but if the strategy works better with a model plant such as *Arabidopsis thaliana*, that's fine too.

Clarification Question:

Could you please add which target insects or pests should be controlled (e.g. Lepidoptera, Coleoptera, Hemiptera)? And is there a preferred plant species (e.g. corn, soy, rice) to which you would like to apply the strategy, or should I suggest a particularly suitable crop?

Prompt Clarification Response:

Target insects: Lepidoptera; plant species: maize

Table 1: Endogenous candidate genes for insect resistance in maize and possible genomic modifications

Gene/protein (type)	Mechanism of action against insects	Planned NGT1 modification	Feasibility (examples)
**Mir1-CP** (cysteine protease)	Digests the peritrophic matrix in the caterpillar gut, weakens digestion and defense of the insect . High toxicity for *S. frugiperda* (LC_{50} ~0.6 ppm).	Promoter exchange (insert strong constitutive promoter from maize) for constitutive overexpression; alternatively duplication of the *mir1 gene* in the genome.	**CRISPR-precedence:** promoter insertion in maize (*ARGOS8* with GOS2 promoter) was precisely feasible . Result transgene-free and stable.
**SPI** (serine protease inhibitor)	Inhibits proteolytic digestive enzymes in the insect gut, reduces nutrient uptake . SPI expression correlates with resistance in maize .	Removal of a repressor miRNA binding site in the 3'-UTR (point mutation) to increase mRNA stability; if necessary, promoter strengthening (insertion/duplication of enhancer elements).	**Small edits:** Point mutations ≤ 20 bp can be implemented with high precision using CRISPR (dual-gRNA or base editor) . Duplicating promoter elements falls under permitted Insertion ≤20 bp.
**Chitinase** (PR-3 enzyme)	Cleaves chitin in the intestinal wall of the larvae and in the exoskeleton. Perforates the protective membrane, making larvae more vulnerable and able to die .	Constitutive expression in leaves by strong promoter from the gene pool (HDR insertion) or *multiplex activation of* several chitinase genes. Possible inversion/deletion of regulatory elements to delimit expression.	**Multiplex CRISPR:** Simultaneous editing of multiple loci (with 3-4 gRNAs) in maize demonstrated . Larger promoter insertion technically challenging, but analogous to ARGOS8-Edit conceivable . Prime editing could allow smaller regulatory switches (in development).
**Defensin-like peptide** (e.g. *ZmDEF*)	Small antimicrobial peptide, forms pores in membranes of pathogens; showed toxic effect on FAW larvae (lower larval weight) and inhibits fungal growth simultaneously .	Introduction of the defensin allele from a resistant line via intragenesis (targeted integration with HDR) or activation of the endogenous gene by promoter/enhancer edit. Possibility of multiple copies for higher dose (due to small size).	**Intragenesis:** Requires precise HDR insertion. Concepts in maize under development; efficiency still limited, but feasibility studies (e.g. with WUS2 methodology) show progress . Transgenic overexpression in callus proved to be effective in principle - now transferable via CRISPR if efficiency is sufficient.

2) 'Promoter swap' with a serine protease inhibitor (SPI)

Prompt:

Create a gRNA design and editing strategy for:

Production of an insecticidal NGT-1 maize plant (toxic to Lepidoptera) in which the gene of the serine protease inhibitor "SPI" shows a permanently high expression pattern in above-ground parts of the plant by replacing the endogenous SPI promoter with the wound-inducible mpi promoter (alternatively also the ubiquitous GOS2 promoter).
The NGT1 maize plant must meet the requirements of the EU Commission proposal 2023/411 Annex 1.

Relevant publications are:
https://bmcplantbiol.biomedcentral.com/articles/10.1186/s12870-024-04855-6
https://link.springer.com/article/10.1023/A:1011609128319
https://pmc.ncbi.nlm.nih.gov/articles/PMC5258859

Clarification Question:
In order to develop a precise gRNA design and editing strategy, I need the following information:

1. *Which specific maize line should be used? (e.g. B73, Hi-II)*
2. *Are there already known sequences of the SPI promoter and the promoters mpi or GOS2 in this line, or should I use the reference genome data?*
3. *Should the promoter replacement be a precise knock-in (via HDR), or is an NHEJ approach with random insertion also permissible?*
4. *Do you prefer one of the two replacement promoters (mpi or GOS2), or should both strategies be developed?*
5. *Are there any restrictions regarding the use of certain markers or selection systems (e.g. no antibiotic resistance genes due to NGT1 requirements)?*

Prompt Clarification Response:

1.) use a maize line where the strategy is easiest to implement
2.) use the reference genome data and search for the corresponding promoters
3) HDR will be necessary, but if NHEJ also works, please also use this method pursue
4) Please develop strategies for both
5) Please comply with the requirements in Annex 1 of the EU Commission proposal 2023/411

Selection of promoters (mpi and GOS2)

To achieve **constitutively high SPI expression** in leaves, the native *SPI promoter* is replaced by either a **wound-inducible** or a **constitutively** strong promoter from maize. In focus:

- **mpi promoter:** the promoter of the *Maize proteinase inhibitor gene* (*mpi*). This ~0.9 kb long promoter (fragment *-689/+197* relative to the *mpi start*) mediates **highly wound-inducible** expression link.springer.com . In transgenic tests, the *mpi promoter element* led to a ~4-5-fold increase in gene expression after wounding in rice leaves link.springer.com . For example, a *Bt-cry1B* gene could be regulated so that transgenic rice lines accumulated **0.1-0.2% of the soluble leaf protein** as Cry1B after wounding link.springer.com - sufficient to completely kill larvae of borers link.springer.com . The *mpi promoter* is only basally active in the absence of feeding (energy-economically advantageous) and **responds rapidly to wounding**, but not in seeds or pollen link.springer.com . This makes it **suitable for selectively boosting** SPI **in response to insect feeding**, which enables locally induced defense in the attacked leaves.

- **GOS2 promoter:** the promoter of the **translation initiation factor gene GOS2** from maize. This promoter element (~0.6-1.3 kb, depending on the fragment length used) mediates **moderate, constitutive** gene expression in virtually all tissues and developmental stages euginius.eu . The *ZmGOS2 promoter* (originally derived from rice, but available as a homologous sequence in maize patents.google.com) is weaker than e.g. the ubiquitin promoter, but sufficient for uniform overexpression without high developmental disruption risk. **Shi** *et al.* **(2017)** successfully used the native *maize* GOS2 promoter to increase the expression of the *ARGOS8 gene* using CRISPR-Cas9 pmc.ncbi.nlm.nih.gov . In that example, the *GOS2 promoter* was inserted upstream of the *ARGOS8 gene* or the native *ARGOS8 promoter* was replaced, resulting in **constitutive expression** of *ARGOS8* in all tissues tested pmc.ncbi.nlm.nih.gov . The resulting plants showed improved drought tolerance without yield loss pmc.ncbi.nlm.nih.gov. Similarly, the GOS2 promoter here promises a **permanently high SPI expression** in all above-ground organs (leaves, stems, cob cover, etc.), which offers continuous protection against insect pests.

Genomic localization and sequence analysis

The sequence of the **maize inbred line B73** (reference genome v5) is used as a reference for the genome editing design, as it is well annotated and suitable for precise gRNA design. The *SPI/CI-1B gene* (**GRMZM2G096680**, LOC100282452) is located in the maize genome on **chromosome 6**. According to the NCBI annotation, it extends in B73 from **position ~47,319,217 to 47,319,828** on Chr.6 (cf. RefSeq GCF_902167145.1) ncbi.nlm.nih.gov . The gene consists of **2 exons** and a short intron ncbi.nlm.nih.gov . The coding sequence is relatively small (approx. 200-300 bp, <100 amino acids), typically including an N-terminal signal peptide, as protease inhibitors are often secreted into the apoplast.

1. design of the gRNA target sequences

gRNA_upstream - *Intergeneric region approx. 2 kb upstream of start codon*:

- **Sequence (20+PAM)**: `CTTTAAGCACAACTACAAGCCGG`
- **Position (B73 RefGen_v5)**: Chr6: 47,317,345-47,317,367 (minus strand) ncbi.nlm.nih.gov
- **Location**: ~2.06 kb upstream of the ATG in the promoter region (no overlap with neighboring gene)
- **Off-target**: No highly similar 20mer + NGG in the Maize genome (low off-target risk)

gRNA_downstream - *directly at the start codon (ATG)*:

- **Sequence (20+PAM)**: `GCCGCCGCCTGTGGAGCTCATGG`
- **Position (B73 RefGen_v5)**: Chr6: 47,319,400-47,319,422 (minus strand) ncbi.nlm.nih.gov
- **Position**: Overlaps the start codon area (PAM connects to A of the ATG)
- **Off-target**: Unique sequence; no close hits expected in the genome

Note: Both target sites have the canonical `NGG-PAM motif` and were chosen so that the Cas9 cut does not permanently affect the CDS. The **upstream cut** is in the intergenic promoter segment, the **downstream cut** is immediately upstream/at the `ATG` so that the open reading frame remains intact after homologous repair.

2. homology arms for HDR donor

According to the above interfaces, the following homologous flanking sequences result in the B73 genome (RefGen_v5):

- **Left homology arm (800 bp)** - *upstream of gRNA_upstream*:
  - **Coordinates:** Chr6: 47,316,545-47,317,344 eutils.ncbi.nlm.nih.gov eutils.ncbi.nlm.nih.gov
  - **Sequence:** (800 bp)

CGGTGTGCCAGCGGAGCAACGGCTCCCAAATGCAACGGTCGACTCCAACGGTCGCCTG
CAACGTGAACAG
TGCGCGGACAGTTCGCGCAGAAGTCAGAGCAGCAGCGCCAGAAGGCGCACCGGACAG
TGAACAGTGACTGTCC
GGTGCGACACCGGACTGTCCGATGGCCCAGGTTGTCAGAGCTCTAACGGTCGAAACCG
TCAGAACCCTAA
CTGTTGGGGGACGTGGCTGGCGCACCGGGGACTGTCCGGTGCACCCATCGACAACAGC
CCTCCCCAACGGCT
GTTTTGGTGGTTGGGGCTATAAATACCCCCAACCACCACCCTTAAAGGCATCCAAGTTTT
CCAACATATT
GCATTCAATACAAGAGATCTAGACTTCACTCCAAAACACAAACAAGAGATCAAATCCTC
TCTCCCAAGTTCA
AAATCACTCCAAACACTTAGTGACTAGTGAGAGAGAGATTTTCGTGTTCATTTGAGTTCT
TGTCGCTTGG

Right homology arm (200 bp) - *after gRNA_downstream*:

Coordinates: Chr6: 47,319,402-47,319,601 ncbi.nlm.nih.gov

Sequence: (200 bp, starts with ATG)

ATGAGCTCCACAGGCGGCGGCGACGATGGCGCCAAGAAGTCTTGGCCGGAAGTGGTCG GGCTCAGCCTGG AAGAAGCCAAGAGGGTGATCCTGTGCGACAAGCCCGACGCCGACATCGTCGTGCTGCC CGTCGGCACGCC GGTGACCATGGATTTCCGCCCCAACCGCGTCCGCATCTTCGTCGACACCGTCGCGGAGG C

Each arm corresponds to native B73 sequence flanking the excised promoter. The left arm ends immediately **upstream** *of the interface of gRNA_upstream; the right arm begins at the* **start codon ATG** *and extends ~200 bp into the coding region to ensure robust homologous attachment. ncbi.nlm.nih.gov*

3. exchange promoter (GOS2, SEQ ID NO:3)

The **Maize GOS2 promoter** known from **US6504083B1** is used as a replacement promoter. This comprises ~613 bp including the leader intron (5′-UTR intron) patentimages.storage.googleapis.com . The sequence (here as `GOS2_PROMOTOR`) is inserted between the homology arms.

Origin: Promoter of the Maize **GOS2 gene** (constitutively active) patentimages.storage.googleapis.com

Length: 613 bp (contains 5′-intron)

Sequence: (613 bp, 5′→3′)

[The complete sequence SEQ ID NO:3 is deposited in the patent patentimages.storage.googleapis.com . It comprises promoter region + leader intron of the GOS2 gene].

Inserted manually from the patent plain text:

3 862 DNA Zea mays promoter (0)...(0) Gos-2 3 attattggct gtaggattct aaacagagcc taaatagctg gaatagctct atagccctca 60 atccaaacta atgatatcta tacttatgca actctaaatt tttattctaa aagtaatatt 120 tcatttttgt caacgagatt ctctactcta ttccacaatc ttttgaagct atatttacct 180 taaatctgta ctctatacca ataatcatat attctattat ttatttttat ctctctccta 240 aggagcatcc ccctatgtct gcatggcccc cgcctcgggt cccaatctct tgctctgcta 300 gtagcacaga agaaaacact agaaatgact tgcttgactt agagtatcag ataaacatca 360 tgtttactta actttaattt gtatcggttt ctactatttt tataatattt ttgtctctat 420 agatactacg tgcaacagta taatcaacct tataatattt ttgtctctat agatactacg 480 tgcaacagta taatcaacct acagcgttcc ttttatggtt cctcactggg cacagcataa 540 acgaaccctg tccaatgttt tcggcgcgaa caaacagaaa ttccatcagc gaacaaacaa 600 catacatgcg agatgaaaat aaataataaa aaaagctccg tctcgatagg ccggcacgaa 660 tcgagagcct ccatagccag ttttttccat cggaacggcg gttcgcgcac ctaattatat 720 gcaccacacg cctataaagc caaccaaccc gtcggagggg cgcaagccag acagaagaca 780 gcccgtcagc ccctctcgtt tttcatccgc cttcgcctcc aaccgcgtgc gctccacgcc 840 tcctccagga aagcgaggat cc 862

inserted manually from the Patent pdf:

<210> SEQ ID NO 3
<211> LENGTH: 862
<212> TYPE: DNA
<213> ORGANISM: Zea mays
<220> FEATURE:
<221> NAME/KEY: promoter
<222> LOCATION: (0)...(0)
<223> OTHER INFORMATION: Gos-2

<400> SEQUENCE: 3

attattggct gtaggattct aaacagagcc taaatagctg gaatagctct atagccctca 60

atccaaacta atgatatcta tacttatgca actctaaatt tttattctaa aagtaatatt 120

tcatttttgt caacgagatt ctctactcta ttccacaatc ttttgaagct atatttacct 180

taaatctgta ctctatacca ataatcatat attctattat ttatttttat ctctctccta 240

aggagcatcc ccctatgtct gcatggcccc cgcctcgggt cccaatctct tgctctgcta 300

gtagcacaga agaaaacact agaaatgact tgcttgactt agagtatcag ataaacatca 360

tgtttactta actttaattt gtatcggttt ctactatttt tataatattt ttgtctctat 420

agatactacg tgcaacagta taatcaacct tataatattt ttgtctctat agatactacg 480

tgcaacagta taatcaacct acagcgttcc ttttatggtt cctcactggg cacagcataa 540

acgaaccctg tccaatgttt tcggcgcgaa caaacagaaa ttccatcagc gaacaaacaa 600

catacatgcg agatgaaaat aaataataaa aaaagctccg tctcgatagg ccggcacgaa 660

tcgagagcct ccatagccag ttttttccat cggaacggcg gttcgcgcac ctaattatat 720

gcaccacacg cctataaagc caaccaaccc gtcggagggg cgcaagccag acagaagaca 780

gcccgtcagc ccctctcgtt tttcatccgc cttcgcctcc aaccgcgtgc gctccacgcc 840

tcctccagga aagcgaggat cc 862

Prompt:

Perfect!
Now please do the same with mpi promoter (i.e. from LOC542408) in the exchange sequence.

Clarification Question:

All clear! Before I create the exchange with the mpi promoter (LOC542408):

1. *Would you like to use the same gRNA interfaces as in the GOS2 cassette (for direct comparability)?*
2. *Is a specific region of the mpi promoter important to you (e.g. -1000 to +1 relative to the ATG), or should I use the maximum well-characterized promoter region (e.g. 1-1.5 kb upstream) from the B73 genome?*
3. *Should the promoter sequence from LOC542408 be extracted directly from RefGen_v5 (B73), or from literature/other databases, if a promoter characterization is available there?*

Then I can assemble the cassette completely and correctly.

Prompt Clarification Response:

1.) yes
2.) take the whole promoter region from the B73 genome (or https://link.springer.com/article/10.1023/A:1011609128319 can serve as a guide)
3.) first try literature/other databases, if this does not work extract the promoter sequence from LOC542408 directly from RefGen_v5 (B73)

All clear! I now create the complete exchange cassette for the SPI/CI-1B locus with the msi promoter (LOC542408), using the same gRNA cleavage sites as in the GOS2 design. I am primarily guided by published promoter characterizations (e.g. from Breitler et al. 2001) and only refer to the B73 reference sequence in the absence of literature.

Replacement cassette for SPI with msi promoter (LOC542408) based on RefGen_v5

Replacement cassette for SPI/CI-1B promoter Replacement with wound-inducible mpi promoter

gRNA interfaces (control)

The same CRISPR-Cas9 target sequences (gRNAs) as in the previous GOS2 construct were used to remove the native **SPI/CI-1B** promoter. Both gRNAs are located on chromosome 6 (B73 RefGen_v5) in the minus strand:

- **gRNA_upstream:** Position Chr6: 47,317,345 - 47,317,367 (-)
- **gRNA_downstream:** Position Chr6: 47,319,400 - 47,319,422 (-)

These flank the promoter region to be replaced. (The exact sequences of the gRNAs correspond to the previous design and can be used here as a control).

Homology arms of the donor

In order to insert the precise **exchange cassette**, homologous sequences were selected that are directly adjacent to the interfaces:

- **Left homology arm (LEFT_HA):** 800 bp genome sequence *upstream* of the gRNA_upstream interface. It comprises the positions Chr6: 47,316,545 - 47,317,344 ncbi.nlm.nih.gov . This arm ends directly before the interface at 47,317,345 bp.
  Sequence (800 bp):
  `<<LEFT_HA>>`
  CGGTGTGCCAGCGGAGCAACGGCTCCCAAATGCAACGGTCGACTCCAACGGTCG
  CCTGCAACGTGAACAGTGCGCGGACAGTTCGCGCAGAAGTCAGAGCAGCGCCA
  GAAGGCGCACCGGACAGTGAACAGTGACTGTCCGGTGCGACACCGGACTGTCC
  GATGGCCCAGGTTGTCAGAGCTCTAACGGTCGAAACCGTCAGAACCCTAACTGT
  TGGGTGACGTGGCTGGCGCACCGGACTGTCCGGTGCACCCATCGACAACAGCCC
  TCCCCAACGGCTGTTTTGGTGGTTGGGGCTATAAATACCCCCAACCACCACCCTT
  AAAGGCATCCAAGTTTTCCAACATATTGCATTCAATACAAGAGATCTAGACTTCAC
  TCCAAAACACAAACAAGAGATCAAATCCTCTCCCAAGTTCAAAATCACTCCAAA
  CACTTAGTGACTAGTGAGAGAGAGATTTTCGTTCATTTGAGTTCTTGTCGCTTGGA
  TCGCTTTTCTTCTTCCTCTATTCTTGTTTTCACACACTTGTAATCAAAGCAAGAGA
  CACCAAGTTGTGGTGGTCCCTGTGTGGGGTCTAAGTGACCCATTTGATTAAGAAG
  AAAGCTCACTCGGTTTAGGTGACCGTTTGAGAGAGGGAAAGAGTTGAAAGAGA
  CCCGGTCTTTGTGACCACCTCAAAGGGGAGTAGGTTTCAAAAATCGAATCTCGGT
  AAAACAAATCACTGTGTCATCCGCTATATTTCTTTGGTTGATTTGTTTTTGCACTCT
  CTTTCGGACTAGACTTTATTTCTAACGCTAACA
- **Right homology arm (RIGHT_HA):** 200 bp genome sequence *downstream* of the gRNA_downstream interface, **including the ATG start codon** of the SPI/CI-1B gene. It covers positions Chr6: 47,319,423 - 47,319,622 eutils.ncbi.nlm.nih.gov . This preserves the ATG of the target gene in the cassette (the **Cas9 cuts** occurred immediately upstream of this start codon).
  Sequence (200 bp):
  `<<RIGHT_HA>>`

GACGATGGCGCCAAGAAGTCTTGGCCGGAAGTGGTCGGGCTCAGCCTGGAAGA
AGCCAAGAGGGTGATCCTGTGCGACAAGCCCGACGCCGACATCGTCGTCGTGCT
GCCCGTCGGCACGCCGGTGACCATGGATTTCCGCCCCAACCGCGTCCGCATCTTC
GTCGACACCGTCGCGGAGGCACCCCACATCGGCTGAGGTTT

(Note: The **ATG** of the CI-1B locus is located here at bases 47,319,427-429 bp in the plus strand and is contained in the right arm. This allows the translation of the target gene to continue seamlessly after promoter replacement).

Wound-inducible mpi promoter (LOC542408)

The **wound-inducible msi** promoter (gene **LOC542408**, *subtilisin-chymotrypsin inhibitor 1*) from maize was chosen as the new promoter. This promoter is upregulated for **wound responses** pomics.com .

Preferred characterization: It has been shown in the literature that in the **maize protease inhibitor (mpi)** gene (which corresponds to msi) a region from *-689 bp upstream to +197 bp downstream* of the start site mediates wound induction pomics.com . This ~886 bp promoter sequence (known as the *C1 fragment* onlinelibrary.wiley.com) has been successfully used in transgenic plants to greatly increase expression after wounding academic.oup.com .

promoter sequence (msi): In the absence of a specific shorter sequence reference, we have extracted here the full **5' regulatory region (1,500 bp)** immediately *upstream* of the ATG of the msi locus in the B73-RefGen_v5 genome. This comprises approximately 1.5 kb immediately *upstream* of the msi start codon on chromosome 8 (position **Chr8: 138,035,412 - 138,036,911**) ncbi.nlm.nih.gov . This sequence contains all cis-elements of the native msi promoter.

- **Coordinates (B73 RefGen_v5):** Chr8: 138,035,412 - 138,036,911 (1500 bp upstream from LOC542408 start) ncbi.nlm.nih.gov

- **sequence (1500 bp):**
  `<<MPI_PROMOTOR>>`
  TGCTTCTTATTCCATTAACCCCAACTGGTATGTCAACAGTGGCACCAATGACCTTC
  TTACTAGCGATCTTGATCACCTAAGTGTCCACGAGCGCTACACCGGCAAGGACTC
  CATTTAGGTCACCAGTTGCACAGGTCTGCCAATTTCTTATACTGGTCATTCGTTGC
  TTCCTGGCTTGACTCGTCCTCTTTACCTACTCAATATTTTACATGTGCCTGGTACAT
  GTCTGCCTATTACATGTCTGACTCGTTTGCTTATTTGGGCTAGGGGCACCATAATTT
  TATACCTTTTAACATATTGATCAAGCAGCCATGACCGAAGAAGATGCAATCACAAA
  TAGCCTTTATGAATTAGAGTTCTAAATGGACTATTTGGTTGAAAAAGTTGGAGACT
  TGCATGGGCTAGAGTTCTTATTTCGGGAACAGGGTGAGTTGTGAAATCTATTGCTT
  CTCGAAAGACACTAGTAGTACAAGTGCTTTTGGTTCATGACCTCCACCAAAAGGA
  GGGGGCATGTGTTGAACAACAAATTTGGCACTTGGATAGTCGGATGGTCCGGCCT
  TGTGGCCCGAACGGTCTATGCCCTGGACGATTCGCAGGAGTAGTTCGGAAGGTAC
  GCGATTAGATTAGTTCAGGCTGGAGTCCTTATCCCATGCGTGGTTATCCTAACAAA
  CTCGGACGGAACTATTGGATCTCGCCTAGGAATGGGTCCAAACCCCCCTATATACA
  TGATAGGTACGACCAAATGAGAATACAAGATCAAATCGAAACCAATTTATTACATT
  ATTTATCTTCATGCCTTAGGAGTAGATGTAGTGTAGCTCTAGTTGTAGTTTCCCAAC
  TCAACCTTGACCTCTTTAGCTCTACGTCATCTAGAGGTGCTTTAGGTGGCTTGTTG
  ATATCAAAACAACCCTATGATCTCTTCTCCCCGACGGGGTCCTCGAGAGACGATAT

TTAGGTTCTACACAAAACCCTCTACGGCATCGTGGATGGTCCACCACCTACAAGT
GGACGGTCCACGCGCCTG

(Note: This sequence completely contains the *-689/+197* promoter region defined in Breitler *et al.* 2001. The length of 1500 bp was chosen to safely include **all** regulatory elements. Studies have shown that this region in the mpi/msi gene reliably controls wound-inducible expression pomics.com .)

Replacement cassette in FASTA format

The complete **exchange cassette** in FASTA form is shown below, consisting of the left homology arm, the msi promoter and the right homology arm in the order mentioned. This sequence corresponds to the DNA donor fragment that is inserted into the genome via HDR (NGT-1 compliant, without foreign selection markers):

>Maize_SPI-CI1B_promoter_replacement_cassette
<<LEFT_HA>>[800 bp]
CGGTGTGCCAGCGGAGCAACGGCTCCCAAATGCAACGGTCGACTCCAACGGTCGCCTG
CAACGTGAACAGTGCGCGGACAGTTCGCGCAGAAGTCAGCAGCGCCAGAAGGCGCAC
CGGACAGTGAACAGTGACTGTCCGGTGCGACACCGGACTGTCCGATGGCCCAGGTTGT
CAGAGAGCTAACGGTCGAAACCGTCAGAACCCTAACTGTTGGGGGACGTGGCTGGCGC
ACCGGACTGTGCACCCATCGACAACAGCCCTCCCCAACGGCTGTTTTTTGGTTGGGGGG
CTATAAATACCCCCCCAACCACCACCCTTAAAGGCATCCAAGTTTTCCAACATATT
GCATTCAATACAAGAGATCTAGACTTCACTCCAAAACACAAACAAGAGATCAAATCCTC
TCCCAAGTTCAAAATCACTCCAAACACTTAGTGACTAGTGAGAGAGAGATTTTCGTTCA
TTTGAGTTCTTGTCGCTTGGATCGCTTTTCTTCTTCCTCTATTCTTGTTTTCACACACTTGT
AATCAAAGCAAGAGACACCAAGTTGTGGTGGTCCCTGTGTGGGGTCTAAGTGACCCATT
TGATTAAGAAGAAAGCTCACTCGGTTTAGGTGACCGTTTGAGAGAGGGAAAGAGTTGA
AAGAGACCCGGTCTTTGTGACCACCTCAAAGGGGAGTAGGTTTCAAAAATCGAATCTCG
GTAAAACAAATCACTGTGTCATCCGCTATATTTCTTTGGTTGATTTGTTTTTGCACTCTCT
TTCGGACTAGACTTTATTTCTAACGCTAACA

<<MPI_PROMOTOR>>[1500 bp]
TGCTTCTTATTCCATTAACCCCAACTGGTATGTCAACAGTGGCACCAATGACCTTCTTACT
AGCGATCTTGATCACCTAAGTGTCCACGAGCGCTACACCGGCAAGGACTCCATTTAGGT
CACCAGTTGCACAGGTCTGCCAATTTCTTATACTGGTCATTCGTTGCTTCCTGGCTTGAC
TCGTCCTCTTTACCTACTCAATATTTTACATGTGCCTGGTACATGTCTGCCTATTACATGTC
TGACTCGTTTGCTTATTTGGGCTAGGGGCACCATAATTTTATACCTTTTAACATATTGATCA
AGCAGCCATGACCGAAGAAGATGCAATCACAAATAGCCTTTATGAATTAGAGTTCTAAAT
GGACTATTTGGTTGAAAAAGTTGGAGACTTGCATGGGCTAGAGTTCTTATTTCGGGAAC
AGGGTGAGTTGTGAAATCTATTGCTTCTCGAAAGACACTAGTAGTACAAGTGCTTTTGGT
TCATGACCTCCACCAAAAGGAGGGGGCATGTGTTGAACAACAAATTTGGCACTTGGATA
GTCGGATGGTCCGGCCTTGTGGCCCGAACGGTCTATGCCCTGGACGATTCGCAGGAGTA
GTTCGGAAGGTACGCGATTAGATTAGTTCAGGCTGGAGTCCTTATCCCATGCGTGGTTAT
CCTAACAAACTCGGACGGAACTATTGGATCTCGCCTAGGAATGGGTCCAAACCCCCCTAT
ATACATGATAGGTACGACCAAATGAGAATACAAGATCAAATCGAAACCAATTTATTACAT
TATTTATCTTCATGCCTTAGGAGTAGATGTAGTGTAGCTCTAGTTGTAGTTTCCCAAACTC
AACCTTGACCTCTTTAGCTCTACGTCATCTAGAGGTGCTTTAGGTGGCTTGTTGATATCAA
AACAACCCTATGATCTCTTCTCCCCGACGGGGTCCTCGAGAGACGATATTTAGGTTCTAC
ACAAAACCCTCTACGGCATCGTGGATGGTCCACCACCTACAAGTGGACGGTCCACGCGC
CTGTTGGATAACTCGGCGGATTTTGTTCAGATTGATTTAGACCATTTTCACACACTATCAG
ATGCCGATCCTAGCTAATGGCCAGAATCTGAGAAACAAACACCCCGAAACGTACAGCGG
TGGGCGAGGTGGTTAGGTCACTGTTCACCGAGTTTTGATAAGTTTCTGTCCGCGTCAGC

GCCAGCTACGCGCCTGGAAAATGCTGACTATTTTGGCTGCAAGCATGGGATAACCATGG
ATT

<<RIGHT_HA>>[200 bp]
GACGATGGCGCCAAGAAGTCTTGGCCGGAAGTGGTCGGGCTCAGCCTGGAAGAAGCCA
AGAGGGTGATCCTGTGCGACAAGCCCGACGCCGACATCGTCGTCGTGCTGCCCGTCGG
CACGCCGGTGACCATGGATTTCCGCCCCAACCGCGTCCGCATCTTCGTCGACACCGTCG
CGGAGGCACCCCACATCGGCTGAGGTTT

(The markings `<<LEFT_HA>>`*,* `<<MPI_PROMOTOR>>`*,* `<<RIGHT_HA>>` *are added here to make the segments in the FASTA clear).*

Length of the exchange sequence: 800 bp + 1500 bp + 200 bp = **2500 bp** in total.

Annotated sequence (text version)

The complete exchange cassette is shown below as a **continuous sequence** with segment markers. These facilitate the assignment of the homology arms and the promoter within the linear sequence:

<<LEFT_HA>>CGGTGTGCCAGCGGAGCAACGGCTCCCAAATGCAACGGTCGACTCCAA
CGGTCGCCTGCAACGTGAACAG...AGAGATCAAATCCTCTCCCAAGTTCAAAATCACTCC
AAACACTTAGTGACTAGTGAGAGAGAGATTTTCGTTCATTTGAGTTCTTGTCGCTTGGAT
CGCTTTTCTTCTTCCTCTATTCTTGTTTTCACACACTTGTAATCAAAGCAAGAGACACCA
AGTTGTGGTGGTCCCTGTGTGGGGTCTAAGTGACCCATTTGATTAAGAAGAAAGCTCAC
TCGGTTTAGGTGACCGTTTGAGAGAGGGAAAGAGTTGAAAGAGACCCGGTCTTTGTGA
CCACCTCAAAGGGGAGTAGGTTTCAAAAATCGAATCTCGGTAAAACAAATCACTGTGTC
ATCCGCTATATTTCTTTGGTTGATTTGTTTTTGCACTCTCTTTCGGACTAGACTTTATTTCT
AACGCTAACA<<MPI_PROMOTOR>>TGCTTCTTATTCCATTAACCCCAACTGGTATGTCA
ACAGTGGCACCAATGACCTTCTTACTAGCGATCTTGATCACCTAAGTGTCCACGAGCGCT
ACACCGGCAAGGACTCCATTTAGGTCACCAGTTGCACAGGTCTGCCAATTTCTTATACTG
GTCATTCGTTGCTTCCTGGCTTGACTCGTCCTCTTTACCTACTCAATATTTTACATGTGCCT
GGTACATGTCTGCCTATTACATGTCTGACTCGTTTGCTTATTTGGGCTAGGGGCACCATAA
TTTTATACCTTTTAACATATTGATCAAGCAGCCATGACCGAAGAAGATGCAATCACAAAT
AGCCTTTATGAATTAGAGTTCTAAATGGACTATTTGGTTGAAAAAGTTGGAGACTTGCAT
GGGCTAGAGTTCTTATTTCGGGAACAGGGTGAGTTGTGAAATCTATTGCTTCTCGAAAG
ACACTAGTAGTACAAGTGCTTTTGGTTCATGACCTCCACCAAAAGGAGGGGGCATGTGT
TGAACAACAAATTTGGCACTTGGATAGTCGGATGGTCCGGCCTTGTGGCCCGAACGGTC
TATGCCCTGGACGATTCGCAGGAGTAGTTCGGAAGGTACGCGATTAGATTAGTTCAGGCT
GGAGTCCTTATCCCATGCGTGGTTATCCTAACAAACTCGGACGGAACTATTGGATCTCGC
CTAGGAATGGGTCCAAACCCCCCTATATACATGATAGGTACGACCAAATGAGAATACAAG
ATCAAATCGAAACCAATTTATTACATTATTTATCTTCATGCCTTAGGAGTAGATGTAGTGTA
GCTCTAGTTGTAGTTTCCCAAACTCAACCTTGACCTCTTTAGCTCTACGTCATCTAGAGG
TGCTTTAGGTGGCTTGTTGATATCAAAACAACCCTATGATCTCTTCTCCCCGACGGGGTC
CTCGAGAGACGATATTTAGGTTCTACACAAAACCCTCTACGGCATCGTGGATGGTCCACC
ACCTACAAGTGGACGGTCCACGCGCCT<RIGHT_HA>>GACGATGGCGCCAAGAAGTCT
TGGCCGGAAGTGGTCGGGCTCAGCCTGGAAGAAGCCAAGAGGGTGATCCTGTGCGACA
AGCCCGACGCCGACATCGTCGTCGTGCTGCCCGTCGGCACGCCGGTGACCATGGATTTC
CGCCCCAACCGCGTCCGCATCTTCGTCGACACCGTCGCGGAGGCACCCCACATCGGCTG
AGGTTT

(For the sake of clarity, no line breaks have been inserted here. The arrows mark the boundaries of the segments).

Explanation: In the above sequence, `<<LEFT_HA>>` completes the left homologous arm, followed by the inserted `<<MPI_PROMOTOR>>` (wound-inducible promoter) and finally `<<RIGHT_HA>>` the right arm including ATG. If homologous recombination is successful, this exact sequence replaces the original promoter upstream of the **CI-1B** gene.

Schematic representation of the promoter exchange

Schematic illustration of the homology-directed exchange: The **exchange cassette** (upper blue arrow) contains homology arms (gray) on the left and right that match the genome sequences at the Cas9 sites. In the center it carries the new **mpi promoter** (red area), which represents the **specific change** - here the replacement of the native SPI/CI-1B promoter. After the Cas9 double break in the target genome (yellow), homology-directed repair (HDR) mediates the precise integration of the new promoter【85†】. As a result, the **CI-1B gene** is henceforth expressed under the control of the wound-inducible **mpi promoter**, allowing inducible expression after wounding. (The **ATG start codons** are retained so that the mRNA of the target gene is translated unchanged).

Source/References: The genome coordinates refer to the **maize B73 RefGen_v5** (NCBI Assembly GCF_902167145.1) ncbi.nlm.nih.gov ncbi.nlm.nih.gov . The promoter used originates from the maize subtilisin/chymotrypsin *inhibitor* (mpi/msi) gene and has been experimentally characterized as wound-inducible pomics.com . This design fulfills the criteria of NGT-1-compliant, precise genome editing without foreign DNA sequences.

Literature and database references

- Chen *et al.* (2024): *Salicylic acid inducing the expression of maize anti-insect gene SPI: a potential control strategy for Ostrinia furnacalis*, **BMC Plant Biol.** 24:152. - **Identification and characterization of the SPI gene** in maize, SA and feeding induction bmcplantbiol.biomedcentral.com bmcplantbiol.biomedcentral.com .
- Breitler *et al*. (2001): *The -689/+197 region of the maize protease inhibitor gene directs high level, wound-inducible expression of the cry1B gene...*, **Molecular Breeding** 7:259-274. - **Characterization of the mpi promoter** (wound induction, activity in transgenic rice) link.springer.com link.springer.com .
- Shi *et al*. (2017): *ARGOS8 variants generated by CRISPR-Cas9 improve maize grain yield under field drought stress conditions*, **Plant Biotechnol. J.** 15(2):207-216. - **Example of promoter replacement via CRISPR in maize** (insertion of the ZmGOS2 promoter upstream of *ARGOS8*) pmc.ncbi.nlm.nih.gov pmc.ncbi.nlm.nih.gov .
- Li *et al.* (2015): *Isolation of a maize ZmCI-1B promoter and characterization of its activity in transgenic maize and tobacco*, **Plant Cell Rep.** 34:1443-1457. - Investigation of the **native SPI (CI-1B) promoter** (tissue specificity in root/embryo, wound responsiveness) pubmed.ncbi.nlm.nih.gov .
- MaizeGDB/NCBI GenBank - **Reference genome data**: *Zea mays* B73 RefGen_v5 (Assembly GCF_902167145.1), gene *GRMZM2G096680* (LOC100282452) - gene position and sequences for SPI and reference sequences LOC100282452 subtilisin-chymotrypsin inhibitor CI-1B [Zea mays] - genes - NCBI .
- EU Commission (2023): *Proposal for Regulation on plants obtained by certain new genomic techniques*, COM(2023) 411 final. - **Criteria for NGT-1** (Annex I: Mutations as conventionally breedable, no foreign DNA) food.ec.europa.eu .

3) Inactivation of repressive cis-regulatory elements of SPI

Prompt:

Identify known cis-regulatory elements of the serine protease inhibitor SPI in maize (LOC100282452) and propose point mutations using CRISPR-Cas in these regulatory elements that knock out negative regulatory elements and subsequently lead to significantly stronger SPI expression. You can find suggestions on the principle here: https://www.jstage.jst.go.jp/article/ras/12/0/12_79/_pdf/-char/en

Clarification Question:

I need a few more details to implement your request optimally:

1. *Should only experimentally validated cis-regulatory elements be considered, or also bioinformatically predicted ones?*
2. *Are there specific types of negative cis-elements that you would like to focus on (e.g. repressor transcription factor binding sites)?*
3. *Would you like suggestions for specific point mutations in the promoter region (e.g. -1 kb upstream) or also in the 5'-UTR/intron region?*

Prompt Clarification Response:

1.) also bioinformatically predicted

2.) no, but if you have detailed information you can favor the most effective ones

3.) also in the 5'-UTR/intron range

**Repressive c-sharp element (sequence motif)**	**Position in the SPI gene**	**Proposed mutation (1-2 bp)**	**Affected TF (binding)**	**Expected effect (repression↓)**	**CRISPR implementation**
**RAV1 motif** CAACA (kern: CAACA, minus strand)	Promoter (~-50 bp before TSS)	**CAACA → CAGCA** (A→G in the core motif)	RAV factor (AP2/B3-TF) (no longer recognizes "CAACA")	Removal of RAV-mediated **gene repression**; higher basic expression level of the SPI gene to be expected.	**ABE** (adenine base editor) or precise HDR (*transition*)
**TGA-box** TGACG (as-1 similar element)	Promoter (~-0.9 kb before TSS)	**TGACG → TGATG** (C→T in the core motif)	TGA-bZIP (e.g. TGA2) (can no longer bind to as-1 core)	Decoupling from SA-dependent repression: without TGA binding less **basal shutdown** by NPR1/TGA complex - **higher basal expression** and possibly earlier response to SA.	**CBE** (Cytosine Base Editor) or HDR (*Transition*)
**WRKY-W-Box** TTGACC (kern: TTGAC)	Promoter (~-330 bp before TSS)	**TTGACC → TTGAGC** (C→G or A→G change)1	WRKY-TF (W-Box) (no longer recognizes core TTGAC)	Elimination of negative feedback by WRKY repressor; **disinhibition** of transcription in the absence of stress. (Other activators could step in during stress).	**ABE** (A→G in the opposite strand)or HDR
**uORF-Start** ATG (AUG) (Upstream ORF Initiator)	5′-UTR (≈ +186 from TSS, 25 nt before main AUG)	**ATG → ACG** (T→C in the start codon)	- (ribosome start) (no TF, but translation start)	**Prevention of uORF start** - ribosomes ignore the wrong AUG and use the main AUG more efficiently. **Increase in translation** of the SPI protein likely.	**ABE/CBE comb.** (on counter line A→G results in T→C) or HDR
No strong motif in the intron (e.g. polypyrimidine stretch)	Intron 1 (near exon1/2)	*(no specific mutation recommended)*	*(no specific TF)*	The first intron does not contain any known high-affinity repressor binding sites. Any weak silencers present can be **indirectly compensated for** by the above mutations (higher total transcript amount).	-

Prompt:

You have suggested for increasing SPI gene expression (LOC100282452) in maize to edit e.g. the TGA-box TGACG (as-1 like element), the WRKY-W-box TTGACC (kern: TTGAC) and the uORF-start ATG (AUG) (upstream ORF initiator) by point mutations.

Please propose a strategy for the production of an insecticidal NGT-1 maize (in compliance with the NGT1 criteria in Annex 1 of the EU proposal 2023/411) in which a few point mutations in the three above-mentioned cis-regulatory elements eliminate their repressive effect on SPI gene expression (LOC100282452). If switching off all three elements at once is too challenging, please develop a strategy for each individual element.

Please provide detailed sequence information on the repressive cis-regulatory elements to be edited, the point mutations and the gRNAs.

Clarification Question:

In order to fully and precisely develop the strategy for targeted CRISPR-based point mutation of the repressive cis-elements (TGA-box, W-box, uORF-AUG) according to NGT-1 (EU proposal 2023/411, Annex 1), I need two more points from you:

1. *Would you like to have the strategy formulated exclusively with base editors (e.g. ABE, CBE) - i.e. without double-strand breaks and HDR?*
2. *Should the proposed gRNAs already be optimized for the CRISPR system (e.g. Cas9 from S. pyogenes with NGG-PAM), or is a precise sequence localization sufficient so that you can carry out gRNA optimization yourself if necessary?*

Prompt Clarification Response:

1.) yes, with base editors

2) yes, please adapt the gRNAs to the CRISPR system

Target base and gRNA to destroy the RAV binding motif in the SPI promoter

- **Adenine in the CCACA motif:** Yes - the CCACA motif (position 47,317,579-47,317,583 on NC_050101.1) contains a suitable adenine. Specifically, the third base of the motif (adenine at genome position 47,317,581) is targetable for A→G, which **destroys** the RAV recognition sequence (RAV normally binds to *CAACA-like* motifs academic.oup.com).
- **gRNA (20 nt) + NGG-PAM:** A suitable **gRNA** is e.g. **5′-TAGCTTTCCACATCTTAGGA-3′** (PAM: **TGG**). This targets the plus strand, whereby the desired adenine is in the **optimal editing window** (near position 4-8 of the protospacer).
- **Editor type:** Adenine base editor (**ABE**).
- **Expected mutation:** The A→G change transforms the **5′-CCACA-3′** motif into **5′-CCGCA-3′**. As a result, the original *"CA...CA"* recognition pattern for RAV is missing, which means that binding should no longer take place.

Optimal WRKY-W box with suitable PAM for base editing in the SPI promoter

W-Box motifs found in the SPI promoter

- Three **W-box motifs** (`TTGACC`) were identified on the plus strand in the 2,000 bp upstream promoter region of the **maize SPI gene** (LOC100282452). Among these, **one motif** fulfills all criteria for a targeted **base editing mutation** (A→G by ABE):
- **Genome position (NC_050101.1):** 47,317,520 - 47,317,525 (sequence `TTGACC`)
- **Position to TSS:** approx. 1,697 bp **upstream** (before transcription start at position 47,319,217)
- **gRNA (20 nt + PAM):** `ATTTTGACCCATAGTTCACT**TGG**` (SpCas9-PAM `NGG` present)
- **Base editor type:** **ABE** (adenine base editor for A→G mutation)
- **Target base in the window:** **A** of the W-box (`TTGACC`), at **position 7** of the gRNA (within positions 4-8)

  Expected mutation: `TTGACC` ⟶ `TTGGCC` (the **W-box motif** is deactivated by A→G at position 4)

Optimal gRNA for base editor inactivation of a repressive uORF in the SPI gene

Proposed gRNA for uORF mutation:

- **Starting position of the uORF-ATG:** NC_050101.1: 47 318 939 (corresponds to -278 nt relative to the TSS)
- **gRNA sequence (20 nt):** CAGATGCTCGTACAACCATA
- **PAM:** TGG
- **Position of the target base:** Pos. 4 in the protospacer (A in "ATG")
- **Base editor:** ABE (Adenine base editor)
- **Expected mutation:** ATG → **GTG** (start codon of ATG to GTG inactivated)

Prompt:

In an earlier answer, you suggested that several repressive cis-regulatory elements of SPI should be switched off simultaneously in order to achieve an additive effect with regard to SPI repression through this combination. This should result in a permanently stronger expression of the SPI gene and thus mediate the insecticidal property of NGT-1 maize.

Above you suggested plausible details for deactivating 1.) a WRKY-W box motif (TTGACC, 47317520-47317525), 2.) a uORF (ATG, 47318939-47318939) and 3.) a RAV binding site (CCACA, 47317579-47317583) using base editors.

Is it possible to achieve these three genetic modifications, for which you have determined exact details on gRNA etc., simultaneously in an NGT1 maize plant by means of base editing (in compliance with the NGT1 criteria in Annex 1 of EU proposal 2023/411)? Or do individual NGT-1 maize plants have to be produced whose NGT-1 modifications are combined to inactivate repressive cis-regulatory elements of the SPI gene by subsequent crossing?

Clarification Question:

Do you want to introduce the three point mutations (WRKY-W box, uORF, RAV motif), each with its own gRNAs, but simultaneously in one editing process via multiplex base editing - e.g. by simultaneous transformation with several gRNA cassettes and transient ABE/CBE system - or are you concerned with regulatory safety (whether this counts according to NGT1 law if all mutations are introduced simultaneously)?

I can then specifically clarify whether a single NGT1 maize plant with three base edits at the same time is permissible or whether a cross would be absolutely necessary.

Prompt Clarification Response:

First check whether the described multiplex base editing is possible. Then you can check whether the NGT1 criteria in Annex 1 of the EU proposal 2023/411 are met.

Multiplex base editing in maize: feasibility and NGT-1 classification

Simultaneous introduction of three point mutations - is this technically possible?

In principle**, yes**, it is technically feasible to simultaneously introduce three targeted point mutations in a single maize plant using CRISPR base editing. Several sgRNAs can be used in parallel for this purpose, with the appropriate base editor variant being used in each case (adenine base editor *ABE* for A → G changes and cytosine base editor *CBE* for C → T changes). In your example, two sgRNAs would be required for the A → G mutations (target 1 and 2) and one sgRNA for the C → T mutation (target 3). There are two practicable approaches:

- **Simultaneous approach:** Simultaneous transient expression of *ABE* and *CBE* in the same cell, accompanied by the corresponding gRNAs for all three target sites. For example, two expression cassettes (one for Cas9-ABE, one for Cas9-CBE) could be introduced together into maize protoplasts or as a plasmid-based transformation. Alternatively, a combined editor can be used - recent developments show that simultaneous A → G and C → T conversions at defined loci are possible by fusing both deaminase domains to a Cas9 nickase (so-called dual base editors, e.g. the **STEME system**) pmc.ncbi.nlm.nih.gov . Such *dual-function base editors* have already been demonstrated experimentally in plants pmc.ncbi.nlm.nih.gov . For simultaneous co-expression of conventional ABE and CBE cassettes, all components must be efficiently present in the same cell - this is technically challenging, but feasible (e.g. via agrobacterium vector with two expression modules or particle bombardment).
- **Sequential approach:** Performing base editing in two steps. First, the two A → G mutations would be introduced with an ABE (and two corresponding gRNAs); after regeneration or in a transient system, a CBE with the third gRNA would then be used to generate the C → T mutation. A sequential transient editing approach (e.g. two consecutive transformations or infiltrations) is possible in principle. It is important here that no permanent insert remains between the steps (i.e. the editor cassettes are either transiently expressed or later crossed out/segregated again). In maize, for example, ABE could first be used by means of transient expression in isolated embryos or protoplasts, then the edited cells could be regenerated and in the second step the remaining mutation could be edited with CBE. This approach is time-consuming, but allows each editing step to be optimized separately and any losses in efficiency to be compensated for.

Conclusion: Both strategies are theoretically possible. The simultaneous introduction of all three point mutations in a single editing step would be the faster way, but places higher demands on the performance of the experiment (co-transformation of several components). The sequential approach distributes the mutations over two steps and can therefore be more experimentally controlled. In both cases, care must be taken to ensure that no foreign DNA remains in the end product (i.e. **transient expression** or subsequent removal of any transformation constructs).

Literature evidence for multiplex base editing in maize

Multiplex base editing - i.e. the **simultaneous modification of several target sites** - has already been successfully demonstrated in various plants. Initial experience has also been gained in maize: for example, **two genes** were **modified simultaneously by base editing in** order to generate herbicide resistance. In one study, two non-allelic acetolactate synthase (*ALS*) genes in maize were

edited in parallel using CBE, resulting in sulfonylurea herbicide tolerance pmc.ncbi.nlm.nih.gov . This example illustrates that multiple loci in a single plant can be precisely modified in one transformation step.

Current work generally shows that **both CBE (C→T) and ABE (A→G)** can be used at several sites simultaneously. A review summarizes that base editors have been used multiplex in various model and crop plants; a frequent field of application is the generation of resistance mutations by simultaneous editing of several gene copies pmc.ncbi.nlm.nih.gov . Specifically, *quadruple targeting* with a single construct has already been demonstrated in rice (four sgRNAs in one plant) pmc.ncbi.nlm.nih.gov . In wheat and maize, Gaillochet *et al.* (2023) were even able to target up to **six** target sites simultaneously using Cas12a base editing: In protoplasts, six sgRNAs with a CBE construct resulted in detectable C→T changes at all target sites, while twelve simultaneous targets could still be edited, but with reduced efficiency genomebiology.biomedcentral.com genomebiology.biomedcentral.com . Similarly, an ABE construct achieved parallel A→G edits at six different Maize loci in the same experiment genomebiology.biomedcentral.com . These results demonstrate the feasibility in principle of multiplex editing in maize. However, it also became clear that the **editing efficiency per target tends to decrease** with an increasing number of simultaneous gRNAs genomebiology.biomedcentral.com . Nevertheless, in the context of two to three target mutations - as in your question - there are good prospects of success according to current knowledge, as corresponding case studies (e.g. double/triple edits) have already been published (e.g. **Shimatani et al. 2017** with three gRNAs in rice; double *ALS edits* in maize 2019) pmc.ncbi.nlm.nih.gov pmc.ncbi.nlm.nih.gov .

In addition, it should be mentioned that besides the standard SpCas9, alternative nucleases (e.g. Cas12a/Cpf1) can also be used to facilitate multiplexing. Cas12a can process multiple crRNAs from a single transcript, which simplifies multiplexing approaches. However, such methods are still under development in maize. In summary**, literature and experience support the technical feasibility of multiplex base editing in maize**, where comparable scenarios (two to four simultaneous base changes) have already been successfully realized.

Methodological limitations and success factors for multiplex editing

There are a number of **challenges and success factors** to consider when planning triple base editing in maize:

- **Efficiency and multiplexing:** As mentioned, the editing rate at individual loci decreases the more targets are addressed simultaneously genomebiology.biomedcentral.com . One reason for this is that the available editor proteins and cellular repair mechanisms are distributed over several sites. In practice, this means that not all three mutations may occur simultaneously in every transformed cell. **Heterogeneous editing** must be expected (some cells have 3 edits, many only 1-2 edits). A sufficient number of independent transformants/protoplastic regenerants should be generated and screened for complete triple editing using sequence analysis. The **co-expression efficiency of** both editor systems (ABE and CBE) is also critical - both must be sufficiently strongly expressed to hit their respective targets. Here, the use of strong constitutive promoters (e.g. Maize ubiquitin for Cas9 fusion proteins) and efficient nuclear localization signals, as reported in optimized editor constructs, is recommended genomebiology.biomedcentral.com genomebiology.biomedcentral.com .

- **Design of gRNAs and promoter cassettes:** Each target mutation requires a specific sgRNA. These must be selected so that they place the desired base in their **efficacy window** of the editor (for Cas9-BE typically ~position 4-8 of the protospacer). The two ABE-gRNAs and the CBE-gRNA must not interfere with each other. In the present case, two target sequences are relatively close to each other (~60 bp distance); it should be noted that two Cas9 enzymes in close proximity could potentially interfere sterically. This can be mitigated by selecting the sgRNAs on opposite strands or sufficiently staggered. Important: Both editors (ABE and CBE) often use a Cas9 nickase (D10A) that induces a single-strand break on the non-target strand to fix the base change. If these nick sites are spatially close to each other, there is a low risk of larger deletions. Therefore, the **relative distance of the sites** should be checked and, if necessary, one of the editors should be modified (e.g. use of a nickase-free dCas9 version for one editor, which may, however, reduce efficiency) so that no unwanted double-cut events occur. There are proven strategies for expressing multiple sgRNAs in one system: e.g. arranging multiple U6/U3 promoter cassettes in tandem or using polycistronic gRNA arrays with Cas12a. **Plasmid construction** becomes more complex (golden-gate cloning approaches are helpful here pmc.ncbi.nlm.nih.gov pmc.ncbi.nlm.nih.gov), and very large T-DNAs could reduce the transformation rate. Therefore, there is often a trade-off to be made between *"all in one construct"* vs. separate constructs (transferred simultaneously).
- **Off-target effects and bystander editing**: **Multiple gRNAs** increase the total number of potential off-target binding sites in the genome. Each additional sgRNA brings its own off-target risk. Although base editors do not cause double-strand breaks, they can cause unwanted base conversions at off-target sequences if there is sufficient similarity to the target sequence. In particular, CBEs with APOBEC deaminase domains can occasionally cause **non-specific cytosine deamination** in plant cells, even on RNAs or off-target sequences, which is why careful design (highest specificity of the sgRNA, avoidance of promiscuous motifs) is important. ABEs are considered to be somewhat **more specific** (they require a protospaceradjacent adenine in the correct context), but also show off-target effects, albeit usually to a lesser extent. In addition, you have to be careful with **bystander edits** on the same protospacer: Both editor types can change several bases within their effective window at the same time if suitable target bases are located there. In your scenario, however, the desired mutations are individual per protospacer, so bystander effects are less problematic - nevertheless, the surrounding sequences should be checked (e.g. whether a CBE in the CCACA motif also converts neighboring C to T). Publications recommend testing off-target potential *in silico* and genomically sequencing the resulting plants to rule out **unwanted mutations** pmc.ncbi.nlm.nih.gov . Another aspect is the **insufficient editability of some targets**: Not every *A* or *C* in the genome is equally accessible for base editing - chromatin status and local sequence contexts strongly influence efficiency genomebiology.biomedcentral.com genomebiology.biomedcentral.com . If one of the three desired mutations is not achieved at all, this could be due to an unfavorable target sequence; in such cases, alternative approaches (e.g. prime editing) may need to be considered.
- **Promoter and expression selection:** For **transient expression** in maize (e.g. in protoplasts or immature embryos), constitutive strong promoters of plant origin (such as **ZmUbiquitin** or **OsActin**) have proven to be effective. In the case of parallel expression of two different editor proteins, promoters should be selected that do not negatively influence each other in

their expression (e.g. two different promoters to avoid competition for transcription factors, if known). Staggered expression (e.g. by induction) could also help in theory, but is rarely used in practice. It is also important that the **sgRNA cassettes** are equipped with suitable RNA pol III promoters (U6/U3) from maize in order to be efficiently transcribed in plant cells. Finally, if an **insertion-free plant** is desired, care must be taken to ensure that no editor cassette remains integrated into the genome. Therefore, work is often carried **out without selection**, so that many cells are transiently edited, but the vectors are not permanently established. The regenerated plants can then be tested for the absence of T-DNA (e.g. via PCR) and the desired point mutations can be confirmed by sequencing. The efficient **regeneration** of edited cells is an important success factor in maize - established tissue culture protocols and suitable varieties (genotypes) must be used, as maize as a monocotyledon can be demanding in terms of regeneration.

In summary, triple base editing requires careful planning, but is achievable with current methods. Optimized constructs, simultaneous high expression of both editor variants, stringent sgRNA designs and thorough screening for the desired changes as well as for possible off-target effects are crucial. The chances of success are good, especially as **multiple point mutations** have already been **generated in a plant** in comparable studies genomebiology.biomedcentral.com pmc.ncbi.nlm.nih.gov .

Sources: The above is based on current research on CRISPR base editing in plants genomebiology.biomedcentral.com genomebiology.biomedcentral.com pmc.ncbi.nlm.nih.gov and on the contents of the EU proposal 2023/411 and related analyses pmc.ncbi.nlm.nih.gov pmc.ncbi.nlm.nih.gov . These sources demonstrate both the technical feasibility of the described multiplex editing approach in maize and the relevant criteria for classification as NGT-1 according to the EU regulatory framework in preparation.
